# The Treatment of Teeth during Pregnancy

**Published:** 1894-02

**Authors:** G. N. Skipp


ARTICLE II.

### THE TREATMENT OF TEETH DURING PREGNNANCY.

BY G. N. SKIPP, L. D. S., ENG.

**Read before the Manchester Odontological Society.**

It is now generally admitted that a considerable change for the worse takes place in the mouth during pregnancy, which is said to be due to constitutional disturbances and the alteration in the normal constituents of the saliva and oral secretions.

It is not my intention to discuss the physiological action of pregnancy on the teeth through the medium of dental pulp and dental tubuli, but rather the external chemical action produced on them by the fluid of the mouth, and the treatment thereof.

I purpose noticing the change in the oral secretions, their effects upon the teeth and temporary filling.

In those cases where pregnancy has any marked action on the teeth we find that it is usually during the *first few months of the first pregnancy* that the oral secretions become so altered, although this is not always the case as it may be put off till the later months, or even deferred till subsequent pregnancies.

When such a condition does occur, we find that the saliva has increased in quantity with an acid reaction; cases being recorded where the flow of saliva has been so great as to imitate Mercurial Ptyalism, which may be owing to irritation of the second and third branches of the fifth pair of nerves, and direct stimulation by acid eructations.

Among the causes of this extra acid condition of the saliva may be mentioned as the three most important:—

I. General disturbance of the digestive organs.

II. Fermentation with the buccal mucus, which has also increased both in quantity and fermentative matter, and particles of food left between the teeth, forming lactic and other acids.

III. Acid eructations from the stomach.

Morning sickness is looked upon by some as a most reliable sign of their pregnancy; previous to which there is an acid taste in the mouth upon awaking. The vomited matter is of a white froth consistency, and so acrid that as soon as it reaches the palate it produces violent retching. This acid vomit has a direct action upon the teeth, rendering them unpleasantly rough, and should there be any superficial caries or facets on their crowns they become very sore during mastication and for some hours after.

Where decay has attacked the teeth to a greater extent, considerable pain is sometimes caused, and patients are glad to plug the teeth with cotton wool in order that the vomit may pass over rather than remain in them.

The important changes in the oral secretions then are:—

I. The saliva has become more acid, which is readily seen by applying litmus paper to the mouth of a patient who is pregnant.

II. The acid saliva has increased both in quantity and fermentive principles.

It is not an uncommon remark for patients to make on learning that the number of fillings required are greatly in excess of what they anticipated:—"I can't understand it; I never had toothache till I was married and then all my teeth seemed to go;" or again, some say, "My teeth never decayed till I was married, and went to live at such and such a place." Not unfrequently the water is blamed, as containing too much iron or being too soft. In most cases, however, the rapid decay which is put down to the water or the change of residence may be easily traced to the causes just enumerated.

In an interesting paper read before this society our President told us that after forty years experience he had come to the conclusion that there was no such thing as either tic douloureux or neuralgia, and that both were either toothache or jaw-ache. I wish some of our medical friends would accept that view, whether it be right or wrong, and send toothache patients to their dentists, instead of treating them to delicious decoctions for the cure of a supposed "neuralgia arising from pregnancy." Many useful teeth might then be saved that otherwise fall victims to want of attention, substituted by a little quinine and iron mixed with a good deal of patience and long suffering.

Although I know of no special form of caries characteristic of pregnancy, there are three kinds of decay which seem more prevalent at this period, and are noted for the rapidity with which they do their work.

1st.—The brown decay in which the surface and margins of the cavity are black; but extending beneath the enamel, and often visible through it, lies a quantity of dark brown softened dentine. This decay attacks the crowns and approximal surfaces of molars and bicuspids, the extent of its ravages depending upon the condition of the fluids of the mouth and the strength of the tooth.

If the tooth structure be well calcified, the area of decay may be limited, seeming merely to bore deeply into the substance to the tooth in the direction of the nerve, which may become exposed, although comparatively little of the tooth has been destroyed.

I remember filling a lady's tooth in this state six weeks after the birth of her first child, and not wishing, as I thought, to come upon the nerve, determined to leave some of the discoloured dentine behind, and after applying creosote and varnishing, the cavity was filled with Weston's cement. The patient returned next morning, having suffered pain during the night, and on removing the filling it was found that a good deal more softened dentine was left than at first supposed and a fine probe passed easily through into the pulp cavity.

If, on the other hand, the tooth structure is poor and imperfectly calcified, the condition is far more serious. Although the decay may at first sight appear merely a small fissure cavity, as soon as it passes the enamel it travels beneath the entire surface, extending upwards to the tops of the cusps and downward to the neck of the tooth,—in fact, infecting the whole tooth above the gum. The colour of the dentine, dark brown to begin with, soon shades off to a pale yellow or straw colour and from that to white, with the layer in immediate contact with the enamel, reduced to a chalky powder. When the tooth is sufficiently excavated to receive a filling nothing but a thin shell of enamel remains.

2nd.—The next form of decay which we find is where a general softening of the tooth takes place without much loss of tissue. The colour remains dark brown all through, and starting from the neck of the tooth on its labial surface, passes round upwards and downwards beneath the enamel and below the gum, which becomes congested and excretes an acrid matter, resembling in odour that of an exposed nerve. The dentine being exceedingly sensitive, both to thermal changes and pressure of the cutting instrument, nitrate of silver is an excellent application for reducing sensitiveness in these cases. The teeth usually affected are lower molars, bicuspids, and upper canines. I have seen a case where an isolated lower bicuspid was considerably raised in its socket from want of antagonism. The decay had passed entirely round the tooth, leaving the enamel comparatively uninjured to the sight, but on cutting it was found to be disintegrated, the whole body of dentine being so softened that a sharp probe might easily have been passed through the pulp. As the tooth was not giving trouble I dosed it with nitrate of silver and left it to its fate.

3rd.—A form of erosion is sometimes present which does not bring about actual caries, but a slight softening with extreme sensitiveness at the necks of the teeth, and facetted points where the enamel is worn away, on the crowns of molars, cutting edges of incisors, etc. To patients wearing artificial teeth this is a great source of trouble. They are unable to bear the slightest pressure from clasps, etc., near the gum edge, and mastication of anything at all solid is a matter of some difficulty. Green tartar or discoloration round the necks of the teeth is associated with the condition of pregnancy, and more so if the patient is of a phlegmatic temperament, when the buccal mucus is secreted in greater abundance—a peculiar sour odor of the breath being also noticed in these patients (who as a rule are not particularly clean in regard to their teeth) and which may be attributed to fermentation.

As a remedy I would suggest keeping the teeth well brushed (especially after eating) with a powder containing soap.

Filling the teeth with metallic filling is not as a rule found to be successful in these cases, cavities carefully cut and filled with amalgam having in a short time developed caries at the edges, especially in approximal cavities of bicuspids, where food has a tendency to lodge.

Osteo fillings disappear like magic, while gutta-percha seems the most useful, being non-irritant, a bad conductor of heat and cold, and not acted upon by acids. It also requires less of the tooth's substance to be cut away to give a firm anchorage, and it is easily removed when a more permanent material can be used.

Jacob's and Hill's preparations are most suitable for this purpose. In accessible cavities, where moisture can be excluded, Jacob's is the best. After drying the cavity thoroughly with absolute alcohol, the gutta-percha is packed in the usual way.

In difficult cavities at the back of the mouth, and in any wet mouth, this is no easy matter, but may be successfully accomplished in the following manner. After excavating the cavity, a piece of Hill's gutta-percha sufficient to nearly fill the cavity is warmed and kneaded between finger and thumb with enough oil of eucalyptus to reduce it to the consistency of thick putty. The cavity is now dried and wiped out with a piece of cotton just moistened with the same oil, and the prepared gutta-percha is then pressed in as quickly as possible with a *cold* instrument. Jacob's gutta-percha may now be pushed in with a warm instrument to bring the filling flush with the edges of the cavity and finished off with chloroform.

The oil of eucalyptus, although a solvent of gutta-percha, evaporates in twenty-four hours and leaves it unimpaired.

Some difficulty is experienced where the gum at the cervical edge bleeds or exudes moisture. This may be obviated by placing over it a small piece of tissue paper saturated in nitrate of silver, and filling the cavity tightly with cotton wool for about ten minutes. On removing the cotton, the tissue paper will adhere to the gum and prevent further trouble, being in turn removed when the filling is finished.

In foul mouths a little iodoform may, if the patient does not object to the smell, with advantage be added in the process of kneading the gutta-percha, but as a rule the antiseptic properties of the eucalyptus are all that is required.

In those cases where the teeth are attacked with the white decay, and it is necessary to give a more solid and unyielding support, the plan adopted is to remove all decay, as far as possible without exposing the nerve, taking care to break down the frail edges of enamel, that the softened dentine may be removed beneath the cusps. The cavity being wiped out with creosote, dried and varnished with Fletcher's copal ether varnish, is filled for about three parts its depth with fossiline to which is added one-fourth its bulk of iodoform, and to the liquid of which a drop of creosote has been added,—Jacob's gutta-percha is packed on the top to complete the filling.

Capped nerves do not answer well during pregnancy; therefore rather than run the risk of exposure, it is better to leave some decayed (disinfectant) dentine behind.

Dead teeth are prone at this period to give trouble, becoming loose and tender or abscessed. If the roots have been filled, the gum above should then be well painted with aconite and iodine, or a capsicum plaster applied at the root. Complete rest should be obtained by removing pressure from the antagonizing tooth.

In teeth where the pulp is but recently dead on giving trouble, the nerve should be removed and the canals cleansed with permanganate of potash, or peroxide of hydrogen, a rhizodontrophy hole drilled, and a filling placed in temporarily, till the patient is in a better state of health to permit more permanent treatment. Should this fail to give relief, and extraction be undesirable, the tooth should be cut down level with the gum and the canals, where accessible, enlarged to give free vent. When extraction is thought necessary, the operator should be alive to the risk and responsibility of producing a miscarriage, and the operation when convenient should be performed at the patient's own house, in the presence of the family doctor, the patient assuming a recumbent position during or immediately after the extraction. Less danger is said to exist when anæsthetics are employed.

Patients are sometimes met with who suffer at the neuralgic foci without sufficient evidence being found in their teeth to account for it, and these we may safely hand over to their medical attendant.

In conclusion, I believe that the rapid decay affecting the teeth during pregnancy, is largely, if not wholly, dependent on the acid condition of the oral secretions; (the teeth, as it were, being constantly subjected to the action of an acid bath of 98°,) and still greater is the effect produced during the hours of sleep, when less saliva is secreted and fermentation becomes more active.

The teeth being unable to withstand an acid solution beyond a certain strength, break down at their most vulnerable points, and the mischief must depend on the resisting power of the tooth structure, and the strength of the acid secretions to which they are subjected.

I can hardly imagine the lime salts being removed through the medium of the pulp and dental tubuli, and more so, as on the authority of Professor Wedl, the pulp becomes subject to fatty degeneration during pregnancy.—*British Journal of Dental Science.*

